# Associations of Dietary Antioxidant and Phytochemical Indices with Cognitive Function: Mediating Roles of Basal Metabolic Rate and Systemic Inflammation

**DOI:** 10.3390/antiox15050573

**Published:** 2026-05-01

**Authors:** Yuebao Fu, Wenjing Wang, Junqiao You, Zijian Cheng, Yuhui Sun, Yijia Liu, Yongye Sun, Yuandi Xi

**Affiliations:** 1 School of Public Health, Qingdao University, Qingdao 266071, China; fuyuebao@qdu.edu.cn; 2 School of Public Health, Capital Medical University, Beijing 100069, China; wangwenjing@mail.ccmu.edu.cn (W.W.); junqiaoyou@163.com (J.Y.); chengzijian199901@163.com (Z.C.); sunyuhui0717@163.com (Y.S.); liuyijia@mail.ccmu.edu.cn (Y.L.)

**Keywords:** composite dietary antioxidant index, dietary phytochemical index, cognitive function, mild cognitive impairment, basal metabolic rate, systemic immune-inflammation index

## Abstract

Dietary antioxidants and phytochemicals are believed to support cognitive health, but evidence on composite dietary indices remains limited. This cross-sectional study of 1845 community-dwelling older adults in China investigated the associations of the composite dietary antioxidant index (CDAI) and dietary phytochemical index (DPI) with cognitive function and mild cognitive impairment (MCI). Cognitive function was assessed using the Montreal Cognitive Assessment (MoCA; Beijing version). MCI was diagnosed through a two-stage procedure: MoCA-based preliminary screening (with education-stratified cutoffs: 13/14 for illiterate, 19/20 for 1–6 years, 24/25 for ≥7 years) followed by neurologist confirmation. CDAI was calculated as the sum of the standardized intakes of six antioxidants (selenium, zinc, carotenoids, vitamin A, vitamin C, vitamin E); DPI was defined as the percentage of the total energy intake from phytochemical-rich foods (fruits, vegetables excluding potatoes, legumes including soy products, nuts, seeds, and whole grains). Multivariable linear regression, logistic regression, and receiver operating characteristic (ROC) curve analyses were performed. The basal metabolic rate (BMR) and systemic immune-inflammation index (SII; platelets × neutrophils/lymphocytes) were tested as potential statistical mediators. Each one-unit increase in CDAI was associated with a 0.068-point higher MoCA score (95% CI: 0.012–0.123), and each one-unit increase in DPI was associated with a 0.029-point higher MoCA score (95% CI: 0.008–0.050). BMR and SII partially mediated the association between CDAI and MoCA score, but temporal ordering remains unclear due to the cross-sectional design. When both CDAI and DPI were in the highest quartile, participants had a 46.3% lower risk of MCI compared with those with both indices in the lowest quartile (OR = 0.537, 95% CI: 0.308–0.935). A predictive model incorporating CDAI, inflammatory markers, and red blood cell parameters showed moderate discriminatory ability in this study sample (apparent AUC = 0.731, bootstrap-corrected AUC = 0.728). These findings suggest that a higher combined dietary antioxidant and phytochemical intake may be jointly associated with better cognitive function, although the cross-sectional design precludes causal inference.

## 1. Introduction

With the continued increase in the aging population, dementia has presented ongoing social and economic challenges [1]. As the most common type of dementia, Alzheimer’s disease (AD) is characterized by progressive cognitive decline and impairment in daily living activities [2]. Mild cognitive impairment (MCI), as the transitional phase between normal aging and dementia, represents a critical opportunity for early diagnosis, prevention and therapy [3]. Given that current medical approaches cannot reverse AD progression, research focus has shifted toward identifying modifiable lifestyle and nutritional factors that may delay cognitive decline in the pre-dementia stage [4].

Emerging evidence suggests that diets that are rich in antioxidants and phytochemicals may support cognitive health [5,6,7]. The composite dietary antioxidant index (CDAI) is a validated composite score derived from the cumulative intake of multiple dietary antioxidants, effectively reflecting dietary antioxidant capacity [8]. Previous cross-sectional studies, including those based on the National Health and Nutrition Examination Survey (NHANES) and the China Health and Nutrition Survey (CHNS), have reported positive associations between CDAI and cognitive performance in older adults [9,10,11,12]. Moreover, the dietary phytochemical index (DPI), defined as the percentage of total energy intake derived from phytochemical-rich foods (fruits, vegetables excluding potatoes, legumes, nuts, seeds, and whole grains) [13], has been less investigated in relation to cognitive function.

Cognitive decline is a complex pathological process involving multiple mechanisms, in which impaired energy metabolism, insufficient oxygen supply, chronic inflammation, and oxidative stress have been shown to play pivotal roles [14,15,16]. In this study, we prioritized two potential intermediary pathways: (1) basal metabolic rate (BMR), which reflects the energy metabolism efficiency and has been linked to cognitive function [17,18]; and (2) the systemic immune–inflammation index (SII, calculated as platelets × neutrophils/lymphocytes), a novel composite inflammatory marker associated with MCI and dementia [19,20]. Based on these considerations, a conceptual pathway linking diet to cognition through basic metabolism and inflammation is plausible. However, given the cross-sectional design, our mediation analyses are exploratory but not causal. In addition to the above potential intermediary indicators, several readily available hematological parameters, including hemoglobin (HGB), mean corpuscular hemoglobin (MCH), and red cell distribution width (RDW), which reflect the oxygen-carrying capacity and erythrocyte homeostasis, have been associated with dementia risk [21], and were included in our analyses as secondary exploratory biomarkers and as candidate predictors for the MCI risk model.

Currently, few studies have simultaneously examined both CDAI and DPI in relation to cognitive outcomes, assessed their joint or comparative effects, or explored the potential pathways linking these dietary indices to cognitive health within a single analytical framework. Therefore, this study aims to investigate the independent and joint associations of CDAI and DPI with cognitive performance and MCI risk in community-dwelling older adults, and to statistically explore whether BMR and SII serve as potential explanatory factors in the association between CDAI and cognitive function. Given the limited availability of existing prediction models for MCI, we also aimed to develop a model incorporating dietary, lifestyle, inflammatory, and erythrocyte parameters.

## 2. Materials and Methods

### 2.1. Participants

**Figure 1:**
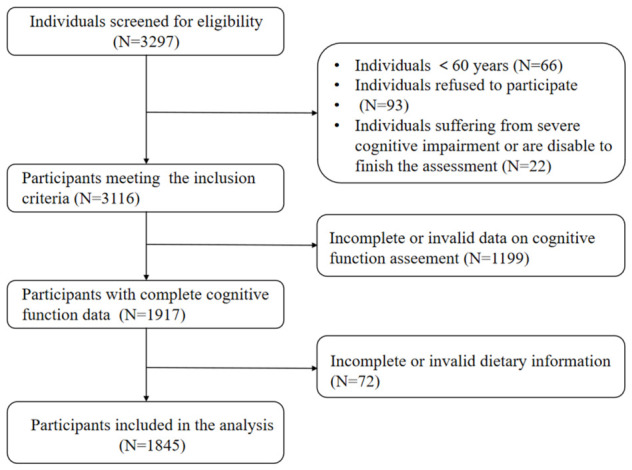
The flowchart of analytic sample selection.

The current study included participants aged 60 years or older from the Effect of Dietary Nutrition on the Cognitive Function and Sarcopenia in Middle-Aged and Elderly People (EDNCS) cohort (Registration number: ChiCTR2100054969, registered on 29 December 2021, available at: https://www.chictr.org.cn/showproj.html?proj=141128, accessed on 27 April 2026). All participants were enrolled through convenience sampling from community health centers in Beijing. Specifically, older adults (≥60 years) visiting these centers for regular health examinations or chronic disease care were invited by trained research staff to join the study. The inclusion criteria were: age ≥ 60 years; normal communication ability that was sufficient to complete a comprehensive interview; and voluntary informed consent to participate. The exclusion criteria included: cognitive impairment caused by depression, traumatic brain injury, or other severe organ dysfunction; refusal to participate in the study; and uncorrected visual or hearing impairment. A face-to-face interview was conducted to assess cognitive function and collect demographic information, chronic disease condition, and dietary information. The present cross-sectional analysis included the baseline participants of the cohort from 2020 to 2023. The study flowchart is presented in Figure 1. Finally, a total of 1845 participants with complete cognitive function assessment and dietary information were included in this study.

The study was conducted in accordance with the Declaration of Helsinki and was approved by the Ethics Committee of Capital Medical University (Approval Nos. Z2019SY052 on 15 October 2019, and 2022SY044 on 9 March 2022). Written informed consent was obtained from all enrolled participants prior to data collection. All personal identifiers were removed from the dataset before analysis, and data were handled in strict compliance with Chinese data protection regulations to ensure participant privacy.

### 2.2. Assessment of Cognitive Function and Definition of MCI

Participants underwent neuropsychological evaluation in a quiet, private room. A survey team of clinical neuropsychologists and research investigators received training on the specifics of the measurements and questionnaires before commencing the examination. Global cognitive function was evaluated using the Mini–Mental State Examination (MMSE) and the Beijing version of the Montreal Cognitive Assessment (MoCA) [22,23]. In the current study, MMSE was used as a screening tool to exclude participants with probable dementia. Based on our previous studies [24], the cut-off point for probable dementia was set at MMSE < 24 (a unified threshold without education stratification). The exclusion was determined by trained research staff (not clinicians) based on the MMSE score; no formal functional assessment was used at this screening stage. Participants were diagnosed with MCI through a two-stage procedure [22,24]. Firstly, a preliminary screening for potential MCI patients was conducted according to the MoCA scores. The education-stratified cutoffs (13/14 for illiterate individuals, 19/20 for 1–6 years of education, and 24/25 for ≥7 years of education) were adopted from previous validation studies in older Chinese adults [23]. No additional education correction points were added to the MoCA scores. Participants who were suspected of having MCI were secondarily examined by neurologists to establish the final diagnosis, based on the following criteria [25]: (1) self-reported concern regarding a change in cognition, assessed using the Subjective Cognitive Decline (SCD) questionnaire; (2) MoCA domain scores indicating impairment in at least one cognitive domain (no additional neuropsychological tests were administered); (3) an Activities of Daily Living (ADL) score indicating preserved functional independence; and (4) not demented.

### 2.3. Dietary Assessment

Dietary information was collected through a food frequency questionnaire (FFQ) comprising 28 food items, adapted from the China National Nutrition and Health Survey (CNHS 2015) [26]. Participants recalled their usual intake over the past year, with frequency options per day, week, month, or year. Portion sizes were estimated using food models and photographic aids. The FFQ was administered face-to-face by trained interviewers using standardized instructions; seasonal foods were averaged across seasons. The energy and nutrient intakes were calculated based on the China Food Composition Database (6th Edition) [27]. Dietary supplements were not included; only food-based intakes were calculated.

### 2.4. Calculation of CDAI

CDAI was assessed according to the method developed by Wright with minor modification [8,28]. The CDAI was calculated based on the daily consumption of 6 antioxidants: selenium, zinc, carotenoids, vitamin A, vitamin C, and vitamin E. To normalize the intakes, we began by subtracting the average intake of each antioxidant and then dividing the outcome by the standard deviation (SD). The calculation formula is as follows:

$$CDAI=\sum \frac{\left(Individual\;intake-Mean\right)}{SD}$$

### 2.5. Calculation of DPI

The DPI was calculated based on the method developed by McCarty on the following formula: DPI  =  daily energy obtained from foods rich in phytochemicals (kcal)/total daily energy intake (kcal)  ×  100 [13]. Foods rich in phytochemicals include fruits, vegetables (except potatoes), legumes (including soy products), nuts, seeds, and whole grains [29]. Potatoes were not considered in the calculations because of their low phytochemical content. The specific food items included in each category are detailed in Table S1.

### 2.6. Assessment of BMR

BMR was estimated by bioelectrical impedance analysis (BIA) (InBody 720 analyzer, InBody Co., Ltd., Seoul, Republic of Korea). Electrodes at the tips of the toes and fingers delivered an electrical current, and body composition was derived from tissue resistance measurements. The BMR values were directly obtained from the device, which estimates BMR using the manufacturer’s proprietary algorithm. Prior to the measurement, participants were instructed to adhere to standard pre-assessment guidelines to minimize potential confounding factors affecting Bioelectrical Impedance Analyzer (BIA) accuracy. Specifically, participants were instructed to: refrain from vigorous exercise for at least 12 h; avoid heavy meals for at least 4 h; void their bladder; remove all metal accessories; and stand quietly for 5–10 min before the test. During the measurement, the participants were instructed to step barefoot onto the weighing platform, with their heels on the rear electrodes and the front part of their feet touching the front electrodes. Meanwhile, they were asked to remain upright while holding the handles to ensure the most accurate measurements possible.

### 2.7. Measurement of Hematological Parameters and Calculation of Blood Inflammation Indices

After an overnight fast (≥12 h), venous blood was drawn and stored according to standard procedures. An automated analyzer measured the following parameters: red blood cell distribution width (RDW); hemoglobin (HGB); mean corpuscular hemoglobin (MCH); and absolute counts of neutrophils, platelets, lymphocytes, and monocytes. The systemic immune–inflammation index (SII) was defined as (P × N)/L and systemic inflammation response index (SIRI) was defined as (N × M)/L, where P, N, M, and L represent the counts of platelets, neutrophils, monocytes, and lymphocytes, respectively [19].

### 2.8. Statistical Analysis

Participant characteristics were presented as the median (interquartile range) for continuous variables due to non-normal distributions, and as the frequency (percentage) for categorical variables. Group comparisons were performed using the Kruskal–Wallis H test or chi-square test, as appropriate.

Multivariate linear regression models were used to assess the associations of the dietary indices (CDAI and DPI) with the MoCA score, and multivariate logistic regression models were employed to evaluate their associations with the risk of MCI. Three incremental models were constructed with progressive adjustment for potential confounders: Model 1 was adjusted for age, sex, and education level; Model 2 was additionally adjusted for body mass index (BMI), smoking status, drinking status, and total energy intake; and Model 3 was further adjusted for chronic diseases including hypertension, diabetes, stroke, and coronary heart disease. Restricted cubic spline (RCS) regression was used to examine dose–response patterns between dietary indices and cognitive outcomes (MoCA score and MCI risk), with adjustment for all covariates in Model 3; four knots were placed at the 5th, 35th, 65th, and 95th percentiles of each dietary index (default in the rms package), and plots were generated using ggplot2 with solid prediction lines and 95% confidence ribbons.

Mediation analysis was conducted to assess the mediating effect of BMR and SII. In the mediation analysis, two models were constructed: a mediator model refers to the linear regression model for evaluating the relationships between the exposure variable and the mediator variables, and an outcome model refers to the linear regression model including both the exposure and mediator variables for assessing their associations with the MoCA score, respectively. The magnitude of the mediating effect was quantified by deriving a mediation percentage, which is calculated as the proportion of the indirect effect to the total effect. The significance of the mediating effect was tested using Bootstrap sampling (times = 5000) [30]. All mediation models were adjusted for the same covariates as in Model 3: age, sex, education, BMI, smoking, drinking, energy intake, hypertension, diabetes, stroke, and coronary heart disease.

Least absolute shrinkage and selection operator (LASSO) regression with 10-fold cross-validation was used to select the predictive factors for MCI. A nomogram was developed to visually represent the prediction model. The model’s discrimination, calibration, and clinical usefulness were evaluated via the area under the curve (AUC), calibration plots, and decision curve analysis (DCA). To correct for optimism, we performed bootstrap resampling (200 iterations). All statistical analyses were performed using SPSS 25.0 and R 4.5.0. A two-sided *p* < 0.05 was considered statistically significant.

## 3. Results

### 3.1. Basic Characteristics by Quartiles of CDAI and DPI

Table 1 presents the basic characteristics of all 1845 participants stratified by quartiles of CDAI and DPI. The median age of the participants was 70 years, and 57.8% were female. Significant differences were observed across the CDAI quartiles for several demographic and lifestyle factors (*p* < 0.05). Participants with higher CDAI were more likely to be male, have an education level of junior high school and above, report current drinking, and have a lower BMI. The prevalence of coronary heart disease also differed significantly across CDAI quartiles. Similarly, characteristics also varied significantly across DPI quartiles (*p* < 0.05). A higher DPI seemed to be associated with a greater proportion of females and lower rates of current smoking and drinking.

### 3.2. Associations of CDAI and DPI with Cognitive Function

#### 3.2.1. Associations of CDAI and DPI with MoCA Score

**Figure 2:**
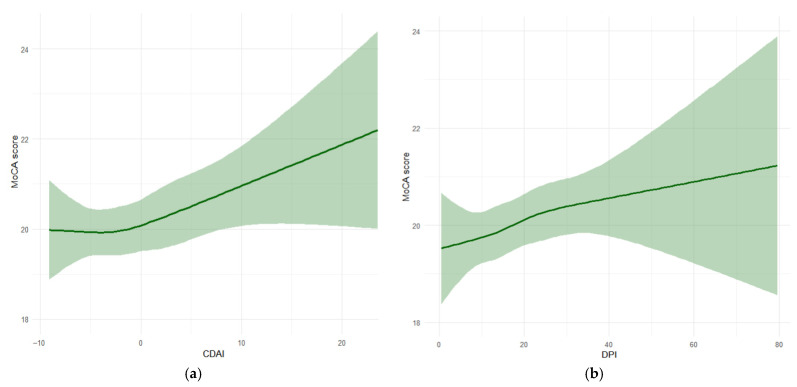
RCS analysis for the dose–response relationship between dietary indices and cognitive function. (**a**) The dose–response relationship between the CDAI and MoCA score, *p* for non-linearity = 0.611 and (**b**) the dose–response relationship between DPI and MoCA score, *p* for non-linearity = 0.883. Models were adjusted for gender, age, education level, BMI, smoking, drinking, energy intake, hypertension, diabetes, stroke and coronary heart disease. Abbreviations: MoCA, Montreal cognitive assessment; CDAI, composite dietary antioxidant index; and DPI, dietary phytochemical index.

Restricted cubic spline (RCS) regression was applied to visualize the dose–response relationships between the dietary indices and cognitive function. After adjusting for all covariates, a positive linear association was observed between both CDAI and DPI and the MoCA score, with no evidence of nonlinearity (*p* for non-linearity = 0.611 and 0.883, respectively) (Figure 2). Multivariate linear regression analyses confirmed these findings, indicating that both higher CDAI and higher DPI were significantly associated with better cognitive performance across all adjusted models (Table 2). In the fully adjusted model, each unit increase in CDAI and DPI was associated with an increase in the MoCA score of 0.068 (95% CI: 0.012–0.123) and 0.029 (95% CI: 0.008–0.050) points, respectively.

To explore the joint associations, participants were categorized by the median values of CDAI and DPI. Compared with the reference group (low CDAI and low DPI), both the “high CDAI and low DPI” group (β = 0.648, 95% CI: 0.049–1.247) and, most prominently, the “high CDAI and high DPI” group (β = 0.775, 95% CI: 0.180–1.370) exhibited significantly higher MoCA scores, suggesting a joint association of the two dietary indices (Table 2).

#### 3.2.2. Associations of CDAI and DPI with MCI

**Figure 3:**
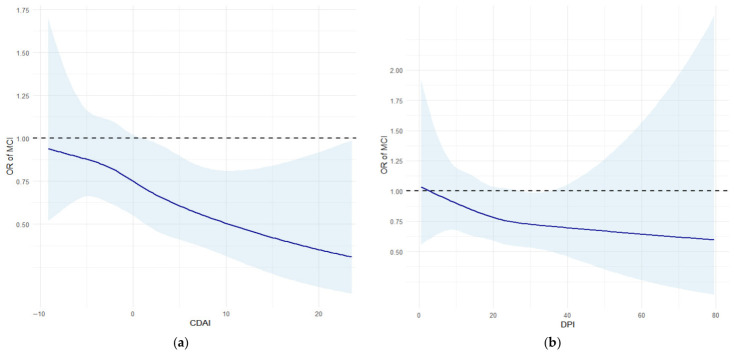
RCS analysis for the dose–response relationship between dietary indices and MCI. (**a**) The dose–response relationship between CDAI and the risk of MCI, *p* for non-linearity = 0.927. (**b**) The dose–response relationship between DPI and risk of MCI, *p* for non-linearity = 0.873. Models were adjusted for gender, age, education level, BMI, smoking, drinking, energy intake, hypertension, diabetes, stroke and coronary heart disease. Abbreviations: MCI, Mild cognitive impairment; CDAI, composite dietary antioxidant index; and DPI, dietary phytochemical index.

The RCS analysis revealed that the risk of MCI decreased linearly with increasing levels of both CDAI and DPI (*p* for non-linearity = 0.927 and 0.873, respectively) (Figure 3). However, while the confidence interval for the CDAI curve fell below the null value (OR = 1) at higher levels, the confidence interval for the DPI curve remained wide and largely included one, indicating a less stable association. Logistic regression models were then used to quantify these associations (Table 3). In the fully adjusted model, a higher CDAI was significantly associated with a lower risk of MCI (OR = 0.964, 95% CI: 0.936–0.992). When CDAI was analyzed as quartiles, participants in the highest quartile (Q4) had a 33.1% lower risk of MCI compared with those in the lowest quartile (OR = 0.669, 95% CI: 0.461–0.972). Although a higher DPI showed a trend toward reduced MCI risk, the association did not reach statistical significance in the continuous analysis nor the quartile-based analysis (both *p* > 0.05).

To explore the joint associations of CDAI and DPI, participants were categorized into three groups based on their quartile distributions: (1) both indices in the lowest quartile (CDAI Q1 and DPI Q1); (2) both indices in the highest quartile (CDAI Q4 and DPI Q4); and (3) all other combinations (composite diet category). Compared with the reference group (CDAI Q1 and DPI Q1), the composite diet category group had a 37.4% lower risk of MCI (OR = 0.626, 95% CI: 0.417–0.941), and the “CDAI Q4 and DPI Q4” group had the greatest reduction in risk, with a 46.3% lower risk (OR = 0.537, 95% CI: 0.308–0.935).

### 3.3. Associations of BMR and Hematologic Parameters with Cognitive Function

In the fully adjusted model, the BMR and HGB levels were positively associated with the MoCA score (β for BMR and HGB were 0.003 and 0.021, respectively) (Table 4). In contrast, elevated SII, SIRI, RDW and MCH were negatively associated with cognitive function (β were −0.001, −0.678, −0.082 and −0.019, respectively) (Table 4).

### 3.4. Associations of Dietary Indices with BMR and Hematologic Parameters

We further examined whether dietary quality influenced these potential mediators (Table 5). In fully adjusted models, higher CDAI was significantly associated with increased BMR and MCH (BMR: β = 2.254, 95% CI: 0.608–3.900; MCH: β = 0.263, 95% CI: 0.001–0.525), while an inverse association was observed with SII (β = −3.700, 95% CI: −7.237 to −0.164). DPI did not show significant associations with these parameters.

### 3.5. Mediating Roles of BMR and SII in the Association of CDAI and MoCA Score

**Figure 4:**
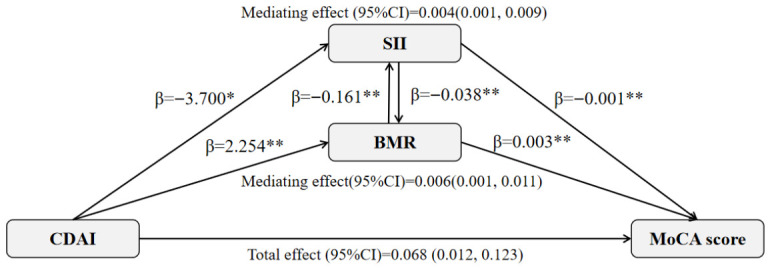
Path diagram of the association between the CDAI and MoCA score with SII and BMR as mediating variables. The mediation proportion was calculated as the ratio of the mediation effect product to total effects: Mediation proportion from BMR = 8.8% and mediation proportion from SII = 5.9%. The mediation models were adjusted for gender, age, education level, BMI, smoking, drinking, energy intake, hypertension, diabetes, stroke and coronary heart disease. * *p* < 0.05, ** *p* < 0.01. Abbreviations: CDAI, composite dietary antioxidant index; MoCA, Montreal cognitive assessment; BMR, basal metabolic rate; and SII, systemic immune–inflammation index.

Given the observed relationships, formal mediation analyses were conducted. As depicted in Figure 4, both the BMR and SII showed statistically significant indirect effects in the association between the CDAI and the MoCA score, accounting for 8.8% and 5.9% of the total effect, respectively. These results are consistent with a hypothesis that a higher dietary antioxidant intake might relate to better cognition through pathways involving a higher BMR and lower systemic inflammation, but causal inference is precluded by the cross-sectional design.

### 3.6. Predictive Model for MCI Based on LESSO Regression

#### 3.6.1. Screening of MCI Predictors and Construction of Nomogram

**Figure 5:**
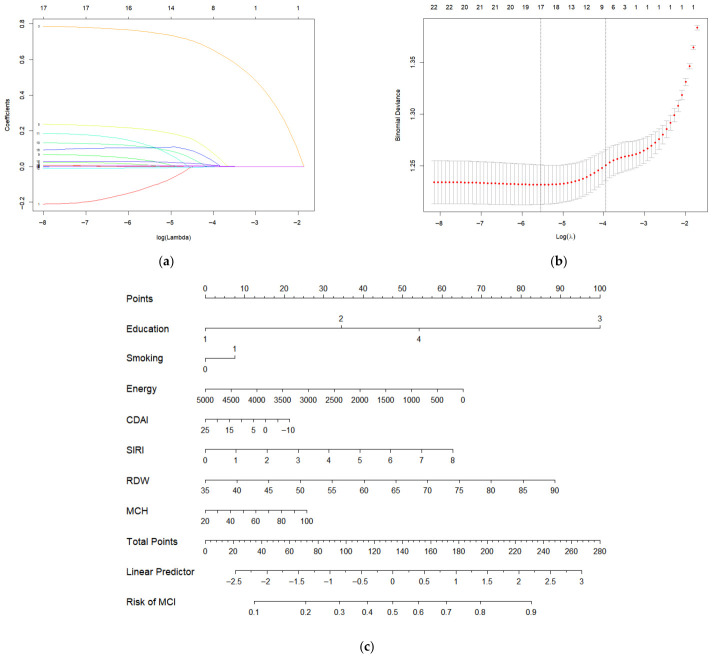
Predictive model for MCI based on LESSO regression. (**a**) The coefficient profiles were generated based on the sequence of log(lambda) and produced non-zero coefficients through the optimal lambda. Each colored line corresponds to a candidate predictor variable in the LASSO model. (**b**) The optimal parameter (λ) in the LASSO model was selected using tenfold cross-validation with the minimum criteria. The two dotted vertical lines respectively indicate the λ value that minimizes the cross-validation error (λmin) and the λ value that gives the most parsimonious model within one standard error of the minimum error (λ1se), which is the final selected model. (**c**) Nomogram for predicting the risk of MCI. The sum of the scores of each predictor corresponds to the risk of MCI. Notes: Education: 1—illiterate; 2—primary school; 3—junior high school; 4—high school or above; Smoking: 0—no; 1—yes. Abbreviations: CDAI, composite dietary antioxidant index; SIRI: systemic inflammation response index; RDW: red cell distribution width; MCH: mean corpuscular hemoglobin; and MCI, mild cognitive impairment.

A multivariate logistic analysis with a LASSO algorithm was used to select potential predictors among dietary quality, BMR, hematological parameters, and demographic characteristics that were associated with MCI (Figure 5a,b). The final model included seven independent predictors: education level, smoking status, energy intake, CDAI, SIRI, RDW, and MCH. Next, a nomogram was developed to visually represent the model (Figure 5c). In the nomogram, each predictor is assigned a point score according to its contribution to the linear predictor, and the sum of the scores of each predictor corresponds to the risk of MCI.

#### 3.6.2. Evaluation of the MCI Predictive Model

**Figure 6:**
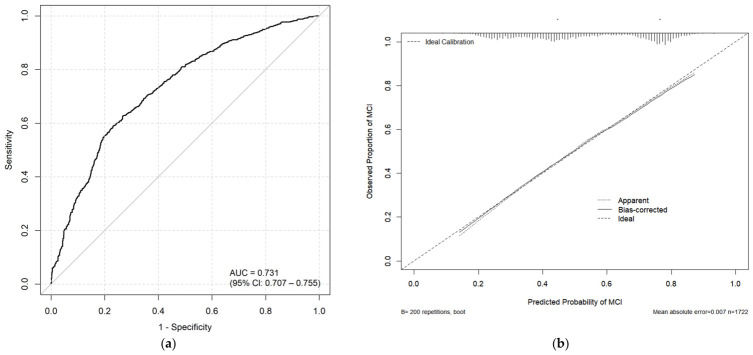
Evaluation of the MCI Predictive Model. (**a**) Receiver operating characteristic (ROC) curve of the predictive model. (**b**) Calibration curve of the predictive model.

**Figure 7:**
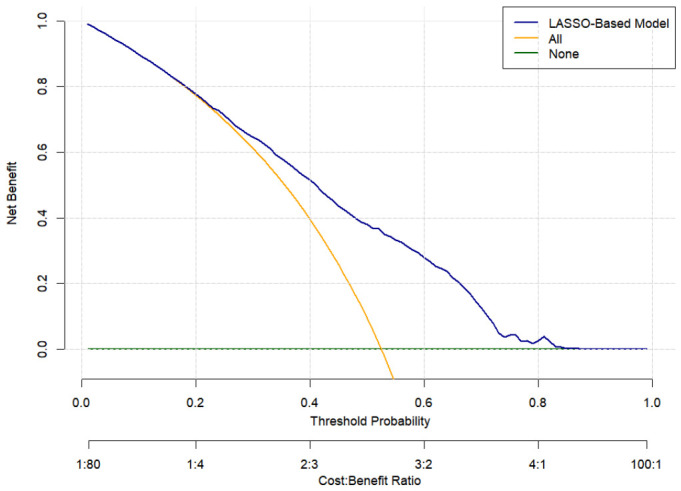
Decision curve analysis (DCA) for the predictive model. The *y*-axis shows the net benefit, and the *x*-axis shows the threshold probability. The blue curve represents the LASSO-based model; the orange curve represents the “treat all” strategy; and the green curve represents the “treat none” strategy.

The apparent area under the ROC curve (AUC) of the LASSO-derived model was 0.731 (95% CI: 0.706–0.756) (Figure 6a). Bootstrap internal validation (200 resamples) yielded an optimism-corrected AUC of 0.728, indicating good discriminative ability without severe overfitting (Table 6). Figure 6b presents the calibration plot of the prediction model. The mean absolute error between the apparent and bias-corrected estimates was 0.007. Bootstrap internal validation further provided an optimism-corrected calibration intercept of 0.079 and slope of 0.971 (Table 6), indicating good overall calibration with a minor tendency to underestimate risk. Decision curve analysis (DCA) showed that the LASSO-based model provided a positive net benefit over a wide range of threshold probabilities (approximately 20–80%), exceeding both the “treat all” and “treat none” strategies (Figure 7). The “treat all” strategy yielded a negative net benefit beyond a threshold of approximately 50%, while the “treat none” strategy gave zero net benefit by definition.

### 3.7. Sensitivity Analyses

To assess the robustness of our findings to the missing data, we performed multiple imputation. The results for CDAI, DPI, BMR, and SII in relation to cognitive outcomes were highly consistent with the complete-case analysis (Tables S4–S6), which suggest that missing data did not materially bias our conclusions.

When CDAI was recalculated using energy-adjusted nutrient intakes (per 1000 kcal), its associations with the MoCA score and MCI risk were no longer statistically significant (Table S7). This discrepancy likely reflects that the original CDAI captures both the quantity and quality of antioxidant intake, whereas energy adjustment isolates density. Given that most published CDAI studies have used the non-energy-adjusted version, we retain the original CDAI as our primary measure and consider the energy-adjusted results to be exploratory.

## 4. Discussion

This study systematically investigated the associations of the composite dietary antioxidant index (CDAI) and dietary phytochemical index (DPI) with cognitive function and mild cognitive impairment (MCI) risk in a community-based middle-aged and older adult population. We further explored the joint associations of these indices, examined the statistically mediating roles of the basal metabolic rate (BMR) and systemic inflammation, and developed a predictive nomogram for MCI. The main findings are as follows: (1) higher CDAI and DPI were independently associated with better cognitive performance; (2) CDAI and DPI showed joint associations with cognitive function, with the most pronounced protection observed in participants with high levels of both indices; (3) BMR and SII were statistically consistent with partial mediation of the association between CDAI and cognitive function; and (4) the LASSO-derived nomogram incorporating CDAI, inflammatory markers, erythrocyte parameters, and demographic factors demonstrated moderate predictive performance for MCI in this study population.

Oxidative stress has been demonstrated to be a vital factor in cellular aging and age-related cognitive decline [15,31]. In this context, increasing studies are focusing on the role of dietary antioxidants in alleviating oxidative stress and promoting cognition [5,32,33]. CDAI provides a composite measure of the total dietary antioxidant capacity, offering a practical tool for public health guidance. Our findings revealed a significant positive correlation between CDAI and overall cognitive function, as well as an inverse relationship with MCI risk. Consistent with our results, several studies based on Chinese populations found a negative correlation between increased CDAI and cognitive impairment [12,34,35]. Zhao et al. similarly identified a significant negative association between CDAI and cognitive impairment in a U.S. population [9]. The convergence of findings across diverse cohorts strengthens the evidence supporting the potential neuroprotective role of the total dietary antioxidant capacity.

In sensitivity analyses, when we recalculated CDAI using energy-adjusted nutrient intakes (per 1000 kcal), the associations with cognitive outcomes were no longer statistically significant. This apparent discrepancy may be explained by the different biological interpretations of the two indices: the original CDAI captures both the absolute quantity and the density of dietary antioxidants, whereas the energy adjustment isolates the density alone. It is possible that in this elderly population, the total energy and nutrient quantity play a more prominent role in cognitive health than the antioxidant density. Notably, most previous studies on CDAI have used the non-energy-adjusted version, and our primary findings are consistent with these studies. Future studies should systematically compare different CDAI calculation methods and consider energy adjustment when appropriate.

The positive association observed between the DPI and cognitive function underscores the potential value of phytochemical-rich diets. Phytochemicals are non-nutritive chemical compounds that are produced by plants whose direct antioxidant properties and consequent health benefits have been well-documented [5,36]. Recent studies have further identified additional potential biochemical and molecular mechanisms, including diverse effects on intracellular and intercellular signaling pathways, such as regulating the nuclear transcription factors and lipid metabolism, as well as modulating the synthesis of inflammatory mediators [37,38]. Moreover, the cognitive-enhancing effects of phytochemicals may also stem from their indirect effects on reducing the harmful factors of age-related cognitive decline, including diabetes, obesity, and cardiovascular disease. A randomized controlled trial in healthy older adults reported that higher flavanol consumption reduced insulin resistance, blood pressure, and lipid peroxidation while improving attention and executive function [39].

Notably, we observed that BMR and SII exhibited statistically mediating effects in the association between the CDAI and MoCA score. Although the underlying mechanisms remain unclear, we can offer potential explanations from the perspectives of energy metabolism and immune inflammation. The brain consumes over 20% of the body’s oxygen, indicating its high sensitivity to energy metabolism disorders [40]. A higher BMR may reflect more efficient systemic metabolism, which could be associated with better cerebral perfusion, mitochondrial function, and ATP production [41,42,43,44,45]. Concurrently, chronic inflammation, particularly innate immune hyperactivity, is implicated in neurodegeneration [46,47,48,49]. The newly proposed SII, integrating platelet, neutrophil, and lymphocyte counts, reflects the balance between innate and adaptive immunity, and has been linked to MCI and dementia [19,49]. One interpretation of our findings is that the inverse correlation between BMR and SII, along with their statistically indirect effects in the diet–cognition relationship, might be explained by crosstalk between metabolic and inflammatory pathways, as well as their combined impact on cognitive function [17,50,51]. This interpretation is consistent with the hypothesis that antioxidant-rich diets enhance mitochondrial integrity, thereby promoting energy metabolic homeostasis, reducing neuroinflammation, and improving cognitive function [52,53,54]. However, given the cross-sectional design, our mediation findings should be interpreted as being associational and exploratory, rather than causal, and they need to be tested in longitudinal or experimental studies.

The LASSO-derived nomogram integrating CDAI, energy intake, SIRI, RDW, MCH, education, and smoking demonstrated a moderate predictive ability for MCI (apparent AUC = 0.731, corrected AUC = 0.728). Moreover, the model utilizes routinely available variables (dietary questionnaires, complete blood counts, demographic information) and is presented in a user-friendly nomogram format, and the positive net benefit for decision curve analysis suggests its potential clinical utility. Nevertheless, this model should be considered hypothesis-generating and internally evaluated; external validation in independent cohorts is required before any clinical application.

Notably, education level showed a complex contribution in the model, with higher education being paradoxically associated with higher risk scores. This finding, while seemingly counterintuitive, aligns with the “compensation hypothesis” proposed by Christensen et al. [55], suggesting that education may provide a cognitive reserve that compensates for the underlying neuropathology, rather than slowing the biological aging process itself. Highly educated individuals may rely more on crystallized intelligence (cognitive reserves) to compensate for potential neuropathological damage; however, when these compensatory mechanisms reach their limits, cognitive decline may be more rapid. However, the cross-sectional design of this study cannot distinguish between the effects of “compensatory maintenance” and “true protection”. Future studies should adopt a longitudinal design and incorporate neuroimaging biomarkers to further elucidate the impact of educational attainment on different cognitive domains and disease stages.

The novelty of this study lies in its separate and joint analysis of CDAI and DPI in relation to cognitive performance and MCI risk, its exploration of BMR and SII as potential explanatory factors in the CDAI–cognition association, and the development of an internally evaluated nomogram for MCI integrating dietary, inflammatory, and hematological factors. Several limitations must be acknowledged upfront to appropriately temper interpretation. Firstly, this is a cross-sectional study, which cannot establish causality. The mediation analyses, in particular, may reflect correlation structures, rather than true temporal pathways. Secondly, dietary intake was assessed using a food frequency questionnaire (FFQ), which is subject to recall bias and measurement error. Thirdly, we did not collect information on dietary supplements, so the calculated antioxidant intakes reflect only food-based sources, which may have led to an underestimation of the total exposure. Fourthly, the exclusion of probable dementia was based on a single MMSE cut-off without education adjustment, which may have misclassified some cases. Fifthly, the associations of DPI with MCI risk were not statistically robust; this instability may reflect the heterogeneity of phytochemical-rich foods or the limitations of DPI as a global measure. Additionally, BMR was estimated using BIA, rather than indirect calorimetry. Although BIA-derived BMR correlates reasonably with measured values, it is subject to prediction error. Finally, several potential confounders were not available, including physical activity, socioeconomic status, depression, sleep quality, APOE genotype, and medication use. Residual confounding from these factors may have influenced the results. Notably, although we adjusted for BMI in the mediation model to account for its potential confounding effect on the BMR–cognition pathway, this adjustment may introduce overadjustment bias if BMI also lies on the causal pathway.

## 5. Conclusions

This cross-sectional study suggests that a higher dietary antioxidant capacity, as measured by CDAI, was associated with a higher MoCA score and lower risk of MCI. A positive association was also observed between the DPI and MoCA score, but the DPI did not show a significant independent association with MCI risk. Notably, we observed joint associations between CDAI and DPI, with the highest MoCA score and lowest MCI risk being observed in participants with high levels of both indices. Mediation analyses were consistent with BMR and SII, partially explaining the association between CDAI and cognition, suggesting potential explanatory pathways involving energy metabolism and systemic inflammation; however, given the cross-sectional design, these findings are exploratory. The nomogram incorporating dietary, inflammatory, and hematological factors demonstrated internally evaluated moderate predictive performance for MCI in this study population. These findings support the hypothesis that combined antioxidant and phytochemical-rich diets may be relevant for cognitive preservation. Future prospective studies are warranted to validate these associations and elucidate causal mechanisms.

## Figures and Tables

**Table 1:** Basic characteristics of participants by quartiles of CDAI and DPI.

Characteristics	Total	Quartiles of CDAI				*p*	Quartiles of DPI				*p*
		Q1	Q2	Q3	Q4		Q1	Q2	Q3	Q4	
Age (years)	70.0 (67.0, 73.0)	69.0 (67.0, 73.0)	70.0 (67.0, 73.5)	70.0 (67.0, 73.3)	69.0 (67.0, 73.0)	0.467	70.0 (67.0, 73.0)	70.0 (67.0, 73.0)	70.0 (67.0, 73.0)	70.0 (67.0, 73.0)	0.822
Gender, n (%)						<0.001					<0.001
Male	778 (42.2)	159 (34.4)	193 (41.9)	193 (41.9)	233 (50.5)		239 (51.8)	204 (44.3)	171 (37.0)	164 (35.6)	
Female	1067 (57.8)	303 (65.6)	268 (58.1)	268 (58.1)	228 (49.5)		222 (48.2)	257 (55.7)	291 (63.0)	297 (64.4)	
Education level, n (%)						0.043					0.392
Primary school and below	984 (53.4)	261 (56.5)	260 (56.4)	224 (48.6)	239 (51.8)		234 (50.8)	244 (52.9)	246 (53.2)	260 (56.4)	
Junior high school and above	861 (46.6)	201 (43.5)	201 (43.6)	237 (51.4)	222 (48.2)		227 (49.2)	217 (47.1)	216 (46.8)	201 (43.6)	
Smoking, n (%)	325 (17.6)	68 (14.7)	73 (15.8)	92 (20.0)	92 (20.0)	0.067	100 (21.7)	91 (19.7)	67 (14.5)	67 (14.5)	0.005
Drinking, n (%)	438 (23.7)	83 (18.0)	101 (21.9)	114 (24.7)	140 (30.4)	<0.001	138 (29.9)	112 (24.3)	94 (20.3)	438 (23.7)	0.001
BMI (kg/m^2^)	26.2 (24.0, 28.6)	26.9 (24.3, 29.1)	26.0 (23.8, 28.6)	25.9 (23.8, 28.6)	26.1 (24.0, 28.3)	0.026	25.9 (23.8, 28.5)	26.2 (23.9, 28.4)	26.0 (24.0, 28.8)	26.6 (24.2, 28.8)	0.406
Hypertension, n (%)	1029 (55.8)	259 (56.1)	266 (57.7)	250 (54.2)	254 (55.1)	0.743	270 (58.6)	259 (56.2)	248 (53.7)	252 (54.7)	0.468
Diabetes, n (%)	452 (24.5)	112 (24.2)	131 (28.4)	102 (22.1)	107 (23.2)	0.129	120 (26.0)	113 (24.5)	123 (26.6)	96 (20.8)	0.166
Stroke, n (%)	84 (4.6)	13 (2.8)	24 (5.2)	19 (4.1)	28 (6.1)	0.097	15 (3.3)	22 (4.8)	18 (3.9)	29 (6.3)	0.138
Coronary heart disease, n (%)	438 (23.7)	83 (18.0)	101 (21.9)	129 (28.0)	125 (27.1)	0.001	101 (21.9)	101 (21.9)	109 (23.6)	127 (27.5)	0.144

Continuous variables were presented as the median (interquartile range), and categorical variables were presented as the number (percentage). Differences between groups were compared by a Kruskal–Wallis test or Chi-square test. Abbreviations: CDAI, composite dietary antioxidant index; DPI, dietary phytochemical index; BMI, body mass index.

**Table 2:** Associations of CDAI and DPI with MoCA score.

	Model 1		Model 2		Model 3	
	β (95%CI)	*P*	β (95%CI)	*p*	β (95%CI)	*p*
CDAI	0.048 (0.006, 0.089)	0.024	0.062 (0.006, 0.117)	0.029	0.068 (0.012, 0.123)	0.017
DPI	0.029 (0.008, 0.050)	0.006	0.029 (0.008, 0.050)	0.007	0.029 (0.008, 0.050)	0.006
CDAI * DPI ^†^						
CDAI low and DPI low	Ref.	-	Ref.	-	Ref.	-
CDAI high and DPI low	0.526 (0.013, 1.040)	0.044	0.635 (0.037, 1.234)	0.037	0.648 (0.049, 1.247)	0.034
CDAI low and DPI high	0.430 (−0.083, 0.943)	0.100	0.440 (−0.080, 0.960)	0.097	0.431 (−0.088, 0.950)	0.103
CDAI high and DPI high	0.633 (0.130, 1.136)	0.014	0.739 (0.149, 1.329)	0.014	0.775 (0.180, 1.370)	0.011

* Different joint combinations of CDAI and DPI. ^†^ CDAI and DPI were categorized into a low level and high level, based on the median values. Model 1: Adjusted for gender, age and education level. Model 2: Additionally adjusted for BMI, smoking, drinking and energy intake in Model 1. Model 3: Hypertension, diabetes, stroke and coronary heart disease were further adjusted in Model 2. Abbreviations: MoCA, Montreal cognitive assessment; CDAI, composite dietary antioxidant index; and DPI, dietary phytochemical index.

**Table 3:** Associations of CDAI and DPI with MCI.

	Model 1		Model 2		Model 3	
	OR (95% CI)	*p*	OR (95%CI)	*p*	OR (95%CI)	*p*
Quartiles of CDAI						
Q1	Ref.	-	Ref.	-	Ref.	-
Q2	0.941 (0.716, 1.239)	0.667	0.975 (0.730, 1.302)	0.863	0.953 (0.712, 1.274)	0.743
Q3	0.794 (0.603, 1.045)	0.099	0.797 (0.568, 1.119)	0.190	0.766 (0.544, 1.080)	0.128
Q4	0.685 (0.520, 0.902)	0.007	0.699 (0.483, 1.012)	0.058	0.669 (0.461, 0.972)	0.035
CDAI continuous	0.966 (0.945, 0.987)	0.002	0.967 (0.939, 0.996)	0.024	0.964 (0.936, 0.992)	0.014
Quartiles of DPI						
Q1	Ref.	-	Ref.	-	Ref.	-
Q2	0.858 (0.653, 1.127)	0.271	0.859 (0.652, 1.132)	0.280	0.858 (0.651, 1.130)	0.276
Q3	0.890 (0.676, 1.171)	0.405	0.870 (0.660, 1.149)	0.327	0.870 (0.659, 1.149)	0.326
Q4	0.816 (0.620, 1.075)	0.148	0.818 (0.620, 1.079)	0.155	0.808 (0.612, 1.068)	0.134
DPI continuous	0.991 (0.980, 1.002)	1.000	0.991 (0.980, 1.002)	0.099	0.990 (0.979, 1.001)	0.088
CDAI * DPI						
CDAI Q1 and DPI Q1	Ref.	-	Ref.	-	Ref.	-
Composite diet category	0.595 (0.402, 0.882)	0.010	0.634 (0.423, 0.951)	0.028	0.626 (0.417, 0.941)	0.024
CDAI Q4 and DPI Q4	0.505 (0.301, 0.847)	0.010	0.559 (0.322, 0.969)	0.038	0.537 (0.308, 0.935)	0.028

* Different joint combinations of CDAI and DPI. Model 1: Adjusted for gender, age and education level. Model 2: Additionally adjusted for BMI, smoking, drinking and energy intake in Model 1. Model 3: Hypertension, diabetes, stroke and coronary heart disease were further adjusted in Model 2. Abbreviations: CDAI, composite dietary antioxidant index; DPI, dietary phytochemical index; and MCI, mild cognitive impairment.

**Table 4:** Associations of BMR and hematologic parameters with MoCA score.

Items	Model 1		Model 2		Model 3	
	β (95%CI)	*p*	β (95%CI)	*p*	β (95%CI)	*p*
BMR	0.002 (0.001, 0.004)	0.001	0.003 (0.001, 0.004)	0.001	0.003 (0.001, 0.004)	0.001
SII	−0.001 (−0.002, −0.0004)	0.006	−0.001 (−0.002, −0.0004)	0.003	−0.001 (−0.002, −0.0003)	0.005
SIRI	−0.700 (−1.114, −0.285)	0.001	−0.703 (−1.122, −0.285)	0.001	−0.678 (−1.098, −0.259)	0.002
RDW	−0.088 (−0.148, −0.028)	0.004	−0.083 (−0.144, −0.022)	0.007	−0.082 (−0.143, −0.021)	0.009
HGB	0.020 (0.005, 0.034)	0.007	0.021 (0.006, 0.035)	0.006	0.021 (0.006, 0.036)	0.006
MCH	−0.019 (−0.029, −0.008)	<0.001	−0.018 (−0.028, −0.008)	0.001	−0.019 (−0.029, −0.008)	<0.001

Model 1: Adjusted for gender, age and education level. Model 2: Additionally adjusted for BMI, smoking, drinking and energy intake in Model 1. Model 3: Hypertension, diabetes, stroke and coronary heart disease were further adjusted in Model 2. Abbreviations: MoCA, Montreal cognitive assessment; BMR, basal metabolic rate; SII, systemic immune–inflammation index; SIRI: systemic inflammation response index; RDW: red cell distribution width; HGB: hemoglobin; and MCH: mean corpuscular hemoglobin.

**Table 5:** Associations of dietary indices with BMR and hematological parameters.

Dietary Indices	BMR		SII		SIRI	
	β (95%CI)	*p*	β (95%CI)	*p*	β (95%CI)	*p*
CDAI						
Model 1	1.783 (0.352, 3.214)	0.015	−1.304 (−3.998, 1.391)	0.343	−0.001 (−0.006, 0.004)	0.737
Model 2	2.015 (0.380, 3.650)	0.016	−0.552 (−7.063, −0.041)	0.047	−0.005 (−0.011, 0.002)	0.155
Model 3	2.254 (0.608, 3.900)	0.007	−3.700 (−7.237, −0.164)	0.040	−0.005 (−0.011, 0.001)	0.123
DPI						
Model 1	0.340 (−0.375, 1.055)	0.351	0.931 (−0.413, 2.274)	0.175	0.001 (−0.002, 0.003)	0.585
Model 2	0.338 (−0.283, 0.959)	0.286	0.838 (−0.502, 2.177)	0.220	0.001 (−0.013, 0.073)	0.668
Model 3	0.388 (−0.233, 1.010)	0.220	0.884 (−0.457, 2.224)	0.196	0.001 (−0.002, 0.003)	0.625
**Dietary Index**	**RDW**		**HGB**		**MCH**	
	**β (95%CI)**	***p***	**β (95%CI)**	***p***	**β (95%CI)**	***p***
CDAI						
Model 1	−0.001 (−0.034, 0.032)	0.957	0.031 (−0.107, 0.169)	0.659	0.255 (0.058, 0.452)	0.011
Model 2	−0.004 (−0.048, 0.040)	0.862	−0.109 (−0.291, 0.072)	0.236	0.238 (−0.023,0.498)	0.073
Model 3	−0.013 (−0.057, 0.031)	0.573	−0.104 (−0.287, 0.079)	0.264	0.263 (0.001, 0.525)	0.049
DPI						
Model 1	−0.002 (−0.019, 0.014)	0.798	−0.060 (−0.129, 0.009)	0.087	−0.080 (−0.179, 0.018)	0.109
Model 2	−0.003 (−0.020, 0.014)	0.709	−0.055 (−0.124, 0.014)	0.116	−0.073 (−0.172, 0.026)	0.149
Model 3	−0.005 (−0.022, 0.012)	0.555	−0.054 (−0.123, 0.016)	0.129	−0.073 (−0.172, 0.026)	0.150

Model 1: Adjusted for gender, age and education level. Model 2: Additionally adjusted for BMI, smoking, drinking and energy intake in Model 1. Model 3: Hypertension, diabetes, stroke and coronary heart disease were further adjusted in Model 2. Abbreviations: CDAI, composite dietary antioxidant index; DPI, dietary phytochemical index; BMR, basal metabolic rate; SII, systemic immune–inflammation index; SIRI, systemic inflammation response index; RDW, red cell distribution width; HGB, hemoglobin; MCH, mean corpuscular hemoglobin.

**Table 6:** Bootstrap internal validation for the MCI predictive model.

	Apparent	Optimism	Corrected
AUC	0.731	0.003	0.728
Calibration Intercept	0	−0.079	0.079
Calibration Slope	1.000	0.029	0.971
Brier Score	0.209	−0.002	0.211

## Data Availability

The data presented in this study are available upon request from the corresponding authors, due to privacy and ethical considerations.

## Supplementary Materials

The following supporting information can be downloaded at: https://www.mdpi.com/article/10.3390/antiox15050573/s1, Table S1: Food items rich in phytochemicals in the DPI calculation; Table S2: Model coefficients and ORs for the LASSO-selected MCI predictors; Table S3: Missing data counts for key variables; Table S4: Associations of dietary indices with MoCA score and MCI after multiple imputation; Table S5: Associations of BMR and hematologic parameters with MoCA score after multiple imputation; Table S6: Associations of dietary indices with BMR and hematologic parameters after multiple imputation; Table S7: Associations of energy-adjusted CDAI with MoCA score and MCI.
