# Supplementary material for: Associations of Dietary Antioxidant and Phytochemical Indices with Cognitive Function: Mediating Roles of Basal Metabolic Rate and Systemic Inflammation
Source: Antioxidants (Basel). 2026 May 1;15(5):573. doi: 10.3390/antiox15050573 (PMC13203818; doi:10.3390/antiox15050573)
Supplement: Supplementary file 1 [file antioxidants-15-00573-s001.zip › antioxidants-4212298-supplementary.pdf]

Supplementary Material

Table S1. Food items rich in phytochemicals in the DPI calculation

Table S2. Model coefficients and ORs for the LASSO-selected MCI predictors

Table S3. Missing data counts for key variables

Table S4. Associations of dietary indices with MoCA score and MCI after multiple imputation

Table S5. Associations of BMR and hematologic parameters with MoCA score after multiple imputation

Table S6. Associations of dietary indices with BMR and hematologic parameters after multiple imputation

Table S7. Associations of energy-adjusted CDAI with MoCA score and MCI

Table S1. Food items rich in phytochemicals in the DPI calculation

Food groups	Food items in the FFQ
Whole grains	Corn, millet, oats, brown rice, coix seeds
Fruits	Apples, pears, peaches, bananas, grapes, oranges, tangerines, watermelons, etc.
Vegetables	Dark-colored vegetables, light-colored vegetables, mushrooms
Nuts	Peanuts, walnuts, pistachios
Seeds	Sunflower seeds, pumpkin seeds, watermelon seeds
Legumes and soy products	Soybeans, black beans, green peas, mung beans, red beans, tofu, soy milk

DPI, dietary phytochemical index

Table S2. Model coefficients and ORs for the LASSO-selected MCI predictors

Variables	Coefficients	ORs
(Intercept)	-2.643	0.071
Education 2	0.763	2.145
Education 3	2.207	9.096
Education 4	1.172	3.228
Smoking1	0.176	1.193
Energy	-0.001	0.999
CDAI	-0.015	0.985
SIRI	0.173	1.189
RDW	0.036	1.037
MCH	0.008	1.008

Notes: Education: 1-Illiterate; 2-Primary school; 3-Junior high school; 4-High school or above; Smoking: 0-No; 1-Yes. Abbreviations: MCI, Mild cognitive impairment; CDAI, composite dietary antioxidant index; SIRI, systemic inflammation response index; RDW, red cell distribution width; MCH, mean corpuscular hemoglobin.

Table S3. Missing data counts for key variables

Variables	Number of valid cases	Number of missing
CDAI	1845	72
DPI	1845	72
BMR	1823	22
SII	1729	116
SIRI	1729	116
RDW	1733	112
HGB	1734	111
MCH	1732	113

CDAI, composite dietary antioxidant index; DPI, dietary phytochemical index; BMR, basal metabolic rate; SII, systemic immune-inflammation index; SIRI, systemic inflammation response index; RDW, red cell distribution width; HGB, hemoglobin; MCH:, mean corpuscular hemoglobin.

Table S4. Associations of dietary indices with MoCA score and MCI after multiple imputation

	MoCA score		MCI	
	β (95%CI)	*P*	OR (95%CI)	*P*
CDAI				
Model 1	0.048 (0.006, 0.089)	0.024	0.966 (0.945, 0.987)	0.002
Model 2	0.060 (0.007, 0.114)	0.030	0.966 (0.938, 0.994)	0.020
Model 3	0.067 (0.013, 0.122)	0.017	0.963 (0.935, 0.991)	0.011
DPI				
Model 1	0.029 (0.008, 0.050)	0.006	0.991 (0.980, 1.002)	0.100
Model 2	0.028 (0.010, 0.046)	0.008	0.991 (0.980, 1.002)	0.108
Model 3	0.029 (0.008, 0.049)	0.007	0.991 (0.980, 1.002)	0.096

Model 1: adjusted for gender, age and education level. Model 2: additionally adjusted for BMI, smoking, drinking and energy intake in Model 1. Model 3: hypertension, diabetes, stroke and coronary heart disease were further adjusted in Model 2. Abbreviations: CDAI, composite dietary antioxidant index; DPI, dietary phytochemical index; MoCA, Montreal cognitive assessment; MCI, Mild cognitive impairment.

Table S5. Associations of BMR and hematologic parameters with MoCA score after multiple imputation

	Model 1		Model 2		Model 3	
	β (95%CI)	*P*	β (95%CI)	*P*	β (95%CI)	*P*
BMR	0.002 (0.001, 0.004)	0.001	0.003 (0.001, 0.004)	0.001	0.003 (0.001, 0.004)	0.001
SII	-0.001 (-0.002, -0.0004)	0.008	-0.001 (-0.002, -0.0004)	0.007	-0.001 (-0.002, -0.0003)	0.011
SIRI	-0.729 (-1.161, -0.297)	0.001	-0.722 (-1.153, -0.290)	0.001	-0.696 (-1.129, -0.263)	0.002
HGB	0.022 (0.007, 0.036)	0.003	0.021 (0.007, 0.035)	0.004	0.021 (0.007, 0.036)	0.004
RDW	-0.091 (-0.153, -0.030)	0.004	-0.091 (-0.153, -0.030)	0.004	-0.089 (-0.151, -0.027)	0.005
MCH	-0.020 (-0.030, -0.010)	<0.001	-0.019 (-0.029, -0.009)	<0.001	-0.020 (-0.030, -0.009)	<0.001

Model 1: adjusted for gender, age and education level. Model 2: additionally adjusted for BMI, smoking, drinking and energy intake in Model 1. Model 3: hypertension, diabetes, stroke and coronary heart disease were further adjusted in Model 2. Abbreviations: MoCA, Montreal cognitive assessment; BMR, basal metabolic rate; SII, systemic immune inflammation index; SIRI: system inflammation response index; RDW: red cell distribution width; HGB:hemoglobin; MCH: mean corpuscular hemoglobin.

Table S6. Associations of dietary indices with BMR and hematologic parameters after multiple imputation

	Model 1		Model 2		Model 3	
	β (95%CI)	*P*	β (95%CI)	*P*	β (95%CI)	*P*
BMR						
CDAI	1.745 (0.316, 3.174)	0.017	2.002 (0.364, 3.641)	0.017	2.241 (0.592, 3.889)	0.008
DPI	0.352 (-0.365, 1.069)	0.336	0.335 (-0.281, 0.951)	0.286	0.387 (-0.230, 1.003)	0.219
SII						
CDAI	-1.155 (-3.912, 1.602)	0.411	-3.460 (-6.979, 0.059)	0.054	-3.553 (-7.099, -0.007)	0.050
DPI	0.821 (-0.542, 2.184)	0.237	0.763 (-0.606, 2.133)	0.274	0.811 (-0.563, 2.185)	0.246
SIRI						
CDAI	0.000 (-0.005, 0.004)	0.841	-0.004 (-0.011, 0.002)	0.187	-0.005 (-0.011, 0.002)	0.163
DPI	0.000 (-0.002, 0.003)	0.719	0.000 (-0.002, 0.003)	0.740	0.000 (-0.002, 0.003)	0.708
HGB						
CDAI	0.033 (-0.113, 0.180)	0.652	-0.105 (-0.287, 0.076)	0.255	-0.098 (-0.282, 0.087)	0.298
DPI	-0.069 (-0.137, 0.002)	0.045	-0.069 (-0.136, -0.001)	0.047	-0.066 (-0.134, 0.001)	0.054
RDW						
CDAI	-0.002 (-0.035, 0.031)	0.910	-0.006 (-0.049, 0.037)	0.786	-0.015 (-0.058, 0.028)	0.502
DPI	-0.001 (-0.018, 0.015)	0.876	-0.002 (-0.018, 0.015)	0.846	-0.003 (-0.020, 0.013)	0.701
MCH						
CDAI	0.261 (0.067, 0.454)	0.008	0.242 (-0.013, 0.496)	0.063	0.267 (0.011, 0.523)	0.041
DPI	-0.087 (-0.188, 0.014)	0.091	-0.080 (-0.180, 0.020)	0.115	-0.078 (-0.178, 0.021)	0.123

Model 1: adjusted for gender, age and education level. Model 2: additionally adjusted for BMI, smoking, drinking and energy intake in Model 1. Model 3: hypertension, diabetes, stroke and coronary heart disease were further adjusted in Model 2. Abbreviations: CDAI, composite dietary antioxidant index; DPI, dietary phytochemical index; BMR, basal metabolic rate; SII, systemic immune inflammation index; SIRI: system inflammation response index; RDW: red cell distribution width; HGB:hemoglobin; MCH: mean corpuscular hemoglobin.

Table S7. Associations of energy-adjusted CDAI with MoCA score and MCI

	MoCA score		MCI	
	β (95%CI)	*P*	OR (95%CI)	*P*
Model 1	-0.049 (-0.108, 0.010)	0.104	1.007 (0.976, 1.039)	0.674
Model 2	-0.054 (-0.113, 0.005)	0.074	1.009 (0.977, 1.041)	0.599
Model 3	-0.052 (-0.111, 0.007)	0.086	1.007 (0.976, 1.040)	0.654

Model 1: adjusted for gender, age and education level. Model 2: additionally adjusted for BMI, smoking, drinking and energy intake in Model 1. Model 3: hypertension, diabetes, stroke and coronary heart disease were further adjusted in Model 2. Abbreviations: CDAI, composite dietary antioxidant index; MoCA, Montreal cognitive assessment; MCI, Mild cognitive impairment.
